# Correlation between refractive errors and ocular biometric parameters at Al-Mustaqbal University, Iraq

**DOI:** 10.1186/s12886-025-04162-0

**Published:** 2025-09-30

**Authors:** Hassan A. Aljaberi, Saeed Rahmani, Amel Muhson Naji

**Affiliations:** 1 Optical Techniques Department, College of Health and Medical Techniques, Al-Mustaqbal University, Babylon, 51001 Iraq; 2 Department of Optometry, Faculty of Rehabilitation, Shahid Beheshti University of Medical Sciences, Tehran, Iran; 3 Department of Optics Techniques, Dijlah University College, Al-Masafi Street, Al-Dora, Baghdad, 00964 Iraq

**Keywords:** Refractive errors, Ocular biometric parameters, Students, Iraq, Cross-sectional study

## Abstract

**Purpose:**

To establish the relationship between ocular biometry and refractive errors in young adult Iraqis by analyzing three critical biometric ocular parameters, including axial length (AL), corneal radius (CR), and central corneal thickness (CCT).

**Methods:**

A cross-sectional study was conducted on individuals aged 18–33 years at Al-Mustaqbal University, Iraq, including 1841 participants (3682 eyes). Quantitative measurements of AL, CR, and CCT were obtained using an Auto Kerato-Refractometer, IOL Master, and pachymetry techniques. Statistical analyses included Pearson correlation, multiple linear regression, one-way ANOVA, and independent samples t-tests to compare biometric parameters between refractive error groups. Generalized Estimating Equations (GEE) were applied to account for the correlation between fellow eyes.

**Results:**

The overall mean AL was 24.45 ± 1.10 mm, mean CR was 7.37 ± 0.77 mm, and mean CCT was 555.83 ± 50.83 μm. Myopic participants had a significantly longer AL (25.11 ± 0.42 mm) compared to hyperopic participants (22.71 ± 0.65 mm; *p* < 0.001). Likewise, myopic eyes had significantly thicker corneas (CCT: 565.62 ± 12.68 μm) than hyperopic eyes (495.42 ± 18.74 μm; *p* < 0.001), as determined by independent samples t-tests. Females exhibited slightly longer ALs than males across both myopic and hyperopic groups (*p* < 0.0001). Regression analysis showed that AL was the strongest predictor of spherical equivalent (SE), followed by CR and CCT. The regression model including AL and CR explained 94.5% of the variance in SE (R² = 0.945).

**Conclusions:**

The findings confirm that AL and CCT are strongly associated with refractive errors, with AL being a primary determinant. This study highlights the role of gender differences in biometric ocular parameters and provides valuable insights into the prevalence of refractive errors in young adults in Iraq. These results can inform future public health initiatives aimed at addressing refractive errors in this population.

**Supplementary Information:**

The online version contains supplementary material available at 10.1186/s12886-025-04162-0.

## Introduction

Biometric ocular measures, including axial length (AL), corneal radius (CR), and central corneal thickness (CCT), are essential in assessing the refractive state of the eye. Variations in these biometric characteristics are strongly associated with an increase of refractive errors, including myopia, hyperopia, and astigmatism. For instance, an elongated axial length is often correlated with myopia, while a shorter axial length typically results in hyperopia. The amount of refractive error is affected by the cornea’s shape and thickness, which in turn affect the retina’s ability to focus light. Based on the prevalence and influence on quality of life, research shows that refractive errors continue to be a major public health concern [1–3]. About 157 million people across the globe have difficulties seeing because their refractive defects have not been corrected, according to the World Health Organization (WHO) [4]. When the eye is unable to properly focus light onto the retina, an inaccurate image is formed, leading to refractive errors [5]. A number of abnormalities in ocular biometric parameters, including axial length, corneal curvature, central corneal thickness, and the lens, contribute to refractive errors such myopia, hyperopia, and astigmatism [5–8]. Research shows that understanding these eye biometric variables is crucial for developing plans to control and avoid refractive errors [9, 10]. Ocular biometrics and refractive errors were reported in several studies focusing on Iran. In Tehran, Hashemi et al. [11] found that the mean axial length was significantly longer in myopic eyes than in emmetropic and hyperopic eyes. The overall prevalence of myopia was estimated to be 21.8% among participants aged 40 to 64, which is an emerging public health problem. Fu et al. [12] reported a myopia prevalence of 4.4% among school-going children; this suggests that geographical and lifestyle factors influence refractive error development.

This has also been furthered by research into the aspects of ocular biometrics related to refractive errors contributed by Mukazhanova et al. [13] documented myopia to be at 25.3% among university students in Kazakhstan, and Wang et al. [14] at 53.7% among high school students in eastern China. The present findings support previous literature regarding the rising prevalence of myopia in the urban and educated populations, as documented by Philip et al. [15] in the analysis of refractive errors in South Indian adults.

The better ophthalmic health infrastructure in Saudi Arabia has enabled it to study myopia in greater detail [16–19]. Wang et al. [14] estimated the prevalence of myopia and reported that 34.5% of high school students in eastern China, are myopic did not wear glasses. Indeed, a significant association of higher axial length was noted, associated with myopic refractive error. Some studies identified urbanization and socioeconomic factors in the variation of the prevalence of refractive errors, observing a higher prevalence in places where near-work activities were more usual in urban settings [15, 20–22].

A comparison of these studies indicates that genetic predisposition factors occur in refractive errors, but environmental and lifestyle factors are essential contributors in the Middle East [23]. The prevalence of myopia indeed differs in neighboring countries, being higher than its rates in other more urbanized regions and where the engagement in near-work activities is higher [24, 25]. Myopic individuals’ mean axial length is invariably more extended than emmetropic and hyperopic individuals; thus, it is an essential biometric marker [26–28].

While numerous studies have explored the relationship between biometric parameters and refractive errors globally, research in the Middle East and particularly Iraq remains limited [18, 29–32]. This gap in knowledge is significant given the unique sociocultural, genetic, and environmental factors in Iraq that may influence the development of refractive errors. To address the rising public health concern of uncorrected refractive defects, shape future therapies, and contribute significant data to the worldwide ophthalmic knowledge base, it is crucial to understand these linkages. Prior research in nearby nations like Saudi Arabia and Iran found strong links between axial length (AL) and refractive errors; variations in these relationships were explained by environmental, genetic, and socioeconomic variables [33–35].

To address this gap, researchers at Iraq’s Al-Mustaqbal University examined the relationship between biometric ocular characteristics and refractive errors in college students and staff. Lifestyle factors, such as restricted outside exposure and prolonged near-work activities, have been associated with the development of myopia, putting university students at a higher risk for refractive errors.

This study will help provide insight on the biometric factors that lead to refractive errors in the Iraqi population by addressing a data gap in the region. Also, it will serve as the groundwork for public health initiatives in the future that aim to control and prevent refractive defects in young people, a group that is becoming more vulnerable as a result of contemporary lifestyle choices.

## Subjects and methods

### Subjects

This study was carried out at Al-Mustaqbal University in Iraq during the 2023–2024 academic year. A total of 1841 subjects were enlisted, encompassing 3682 eyes, with ages ranging from 18 to 33 years. The majority of participants were females, and males comprised 42.23% (*n* = 1555) of the total sample. The overall mean age was 22.21 ± 3.41 years.

The sample size (*n* = 3682 eyes) was determined based on an a priori statistical power analysis following standard methodology. The analysis aimed to detect a moderate effect size (Cohen’s d = 0.5) with 80% statistical power and a two-tailed significance level of 0.05. The standard deviations (SDs) for ocular biometric parameters (e.g., axial length, central corneal thickness) were derived from pilot data and previous studies, and were used as key inputs in the sample size estimation. A moderate effect size was selected based on the expected magnitude of biometric variation between refractive error categories. The relatively large sample size enhances the robustness, statistical reliability, and representativeness of the findings in this university-based population. While the sample was drawn from a university population, this group represents a critical demographic where refractive errors are increasingly prevalent due to environmental and lifestyle factors. Nonetheless, we acknowledge that the sample may not fully capture the diversity of the broader Iraqi young adult population. This limitation is noted in the discussion, and future research should include broader geographic and socioeconomic representation to enhance generalizability.

Participants were selected based on clear inclusion and exclusion criteria. All subjects were required to be students or employees at Al-Mustaqbal University at the time of data collection. Participants with a history of ocular surgery, systemic diseases (e.g., diabetes), ocular trauma, or unrelated eye conditions were excluded. Only individuals with stable ocular health were included to avoid factors that could affect refractive outcomes.

**Fig. 1:**
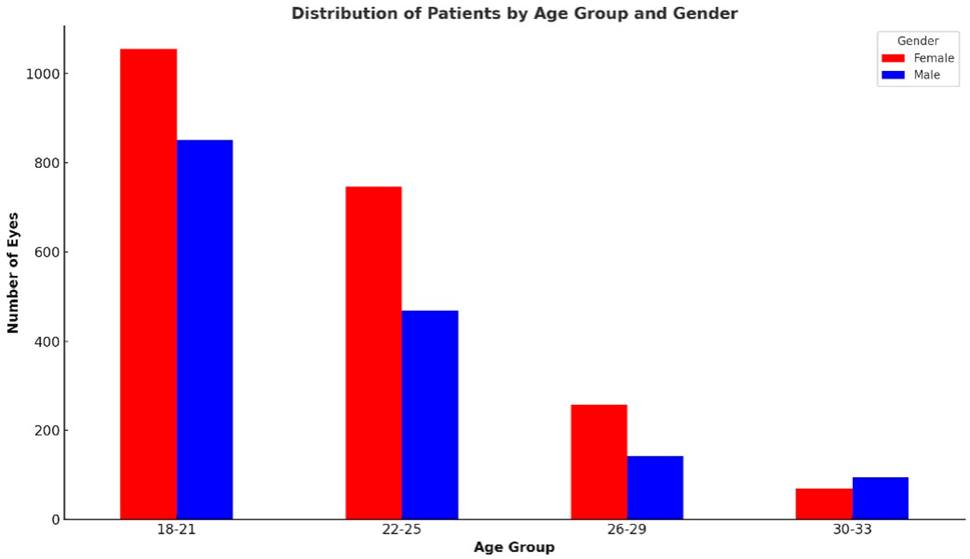
Distribution of participants’ eyes by age group and gender

Figure 1 shows the distribution of participants’ eyes by age group and gender. Younger individuals, particularly in the 18–21 and 22–25 age groups, were more represented, which is reflective of typical university demographics. This trend aligns with typical university demographics, where female participation may be higher in certain disciplines. Separating participants by gender and age helps reduce bias and facilitates more accurate comparisons of biometric measurements across different groups.

### Examinations and assessments

Detailed ophthalmologic examinations were conducted on all 1841 individuals, resulting in a total of 3682 eyes examined. Refraction was assessed using automated refraction followed by subjective refinement to determine the spherical equivalent refractive error for each eye. Axial length (AL) was measured using a non-contact IOL Master, which provides high-precision ocular biometry. Corneal curvature was measured using the Topcon KR-800 Auto Kerato-Refractometer. Central corneal thickness (CCT) was measured using an ultrasonic pachymeter, which was specifically selected due to its clinical accuracy, portability, and proven reliability across diverse patient groups. While the IOL Master does offer a corneal thickness measurement function, in our setting it was not available with the upgraded CCT module. Furthermore, the ultrasonic pachymeter has been validated in multiple studies for precise corneal thickness assessment and remains a gold-standard method in many clinical contexts. The devices used were the latest models available in our university laboratories and were regularly calibrated and maintained by certified optometrists and ophthalmologists to ensure measurement precision. This method allowed us to capture accurate biometric data suitable for robust statistical analysis.

Furthermore, anthropometric measurements, encompassing ocular biometrics, were collected, succeeded by additional assessments to investigate the relationships between ocular dimensions and other ophthalmological parameters pertinent to refractive errors. This thorough methodology offered a detailed perspective on the ocular health status of the study population.

### Definitions

Refractive error (RE) is categorized according to the spherical equivalent (SE), ascertained by aggregating the spherical value with half of the cylindrical value. Myopia or nearsightedness is defined by a spherical equivalent (SE) of ≥ -0.50 diopters (D). Mild to moderate myopia is defined as ranging from − 0.50D to -6.00D, but severe myopia is characterized by a spherical equivalent above − 6.00D. Hyperopia, or farsightedness, is defined by a spherical equivalent (SE) of ≥ + 0.50 diopters (D). Astigmatism is defined as a cylindrical refractive error of ≥ 0.50 diopters, irrespective of the spherical equivalent. These classifications align with recommendations from prominent ophthalmologic conferences and generally recognized research.

Axial length (AL) is the measurement from the corneal surface to the retina, significantly influencing refractive errors. An elongated axial length (AL) is linked to myopia, whereas a shortened AL is connected with hyperopia. The corneal radius (CR) denotes the curvature of the cornea; anomalies in CR are associated with astigmatism and other refractive disorders. center corneal thickness (CCT) quantifies the thickness of the cornea’s center region and is crucial for evaluating intraocular pressure and refractive disorders, including glaucoma.

### Data analysis

Statistical analyses were conducted using SPSS version 26.0 (SPSS Corp., Chicago, USA). Objective measurements for spherical equivalent (SE), axial length (AL), corneal radius (CR), and central corneal thickness (CCT) were collected for both eyes of all participants.

The Kolmogorov–Smirnov test was employed to evaluate the normality of distribution for SE, AL, CR, and CCT across male and female participants. A significance level of *p* < 0.05 was considered indicative of non-normality, and appropriate non-parametric methods were applied where this assumption was violated. To compare SE and AL between males and females, an independent samples t-test was applied, as the two groups are independent.

Associations between biometric variables were assessed using Pearson correlation coefficients, and further examined using multiple linear regression analysis. These models were developed to investigate the predictive influence of AL, CCT, and CR on SE, with analyses stratified by gender to identify potential sex-based interactions.

Given that both eyes from each participant were included in the dataset, the inherent correlation between fellow eyes was accounted for using Generalized Estimating Equations (GEE). This method ensures valid statistical inference by adjusting for within-subject correlation in bilateral data.

To assess differences in AL, CR, and CCT across refractive error categories (myopia, hyperopia, and astigmatism), a one-way analysis of variance (ANOVA) was conducted. Where significant differences were observed, Least Significant Difference (LSD) post hoc tests were used to determine pairwise group differences.

For inter-group comparisons, a stricter significance level (*p* < 0.001) was applied to reduce the likelihood of detecting false-positive results that may arise when performing multiple statistical tests. All data are presented as mean ± standard deviation (SD) and 95% confidence intervals (CI), unless otherwise stated.

Finally, to support and visualize quantitative findings, scatter plots were generated for key relationships, each annotated with the corresponding regression equation and R² value, thereby enhancing the interpretability of predictive trends among biometric parameters.

## Results

### Biometric parameters and refractive error distribution

The mean axial length (AL) for all individuals was 24.45 ± 1.10 mm, the mean corneal radius (CR) was 7.37 ± 0.77 mm, and the mean central corneal thickness (CCT) was 555.83 ± 50.83 μm. Table 1 delineates the average ocular biometric data categorized by type of refractive error. Deviations in corneal curvature in myopic and hyperopic patients presumably arise from a confluence of genetic, environmental, developmental, and measurement-related factors. These variances are crucial for comprehending the complete intricacy of refractive defects and their clinical therapy. The clinical interpretation of refractive error data is constrained without a thorough examination of the interaction between corneal curvature and axial length in these patients.

**Table 1:** Mean ocular biometric measurements stratified by refractive error type, including AL, CR, and CCT

Refractive Error	*N*	AL Mean ± SD (mm)	CR Mean ± SD (mm)	CCT Mean ± SD (µm)
Myopia	1871	25.11 ± 0.42	6.86 ± 0.44	565.62 ± 12.68
Hyperopia	626	22.71 ± 0.65	8.53 ± 0.38	495.42 ± 18.74
Astigmatism	1185	24.31 ± 0.96	7.54 ± 0.58	530.93 39.94

AL– Axial Length, CR– Corneal Radius, CCT– Central Corneal Thickness, SD– Standard Deviation

### Correlation and multiple regression analyses

**Fig. 2:**
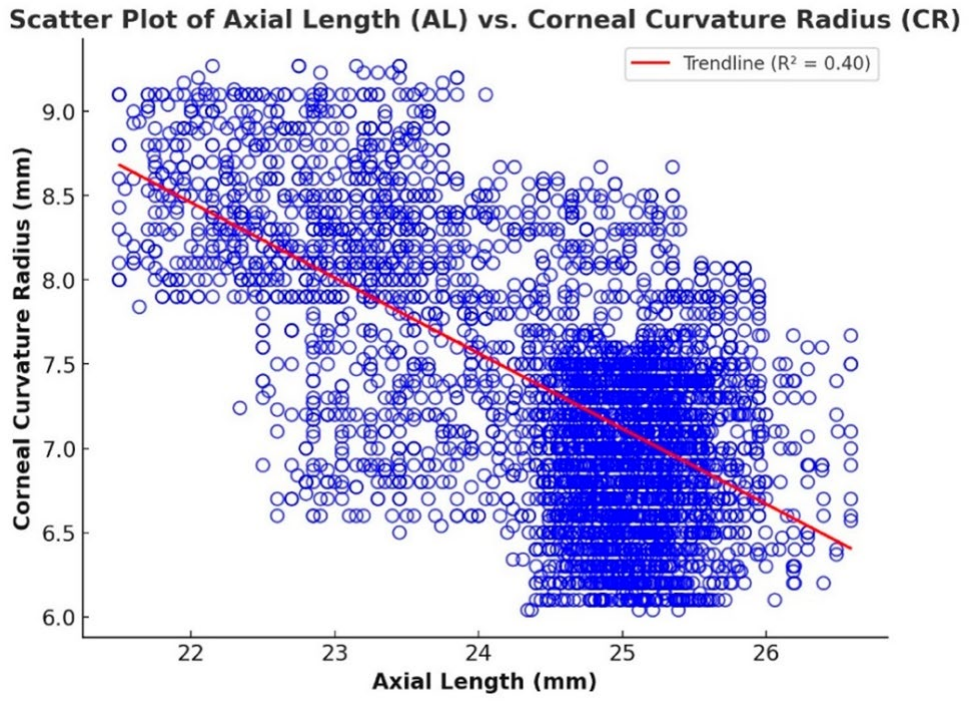
Scatter plot showing a correlation between axial length and corneal radius

**Fig. 3:**
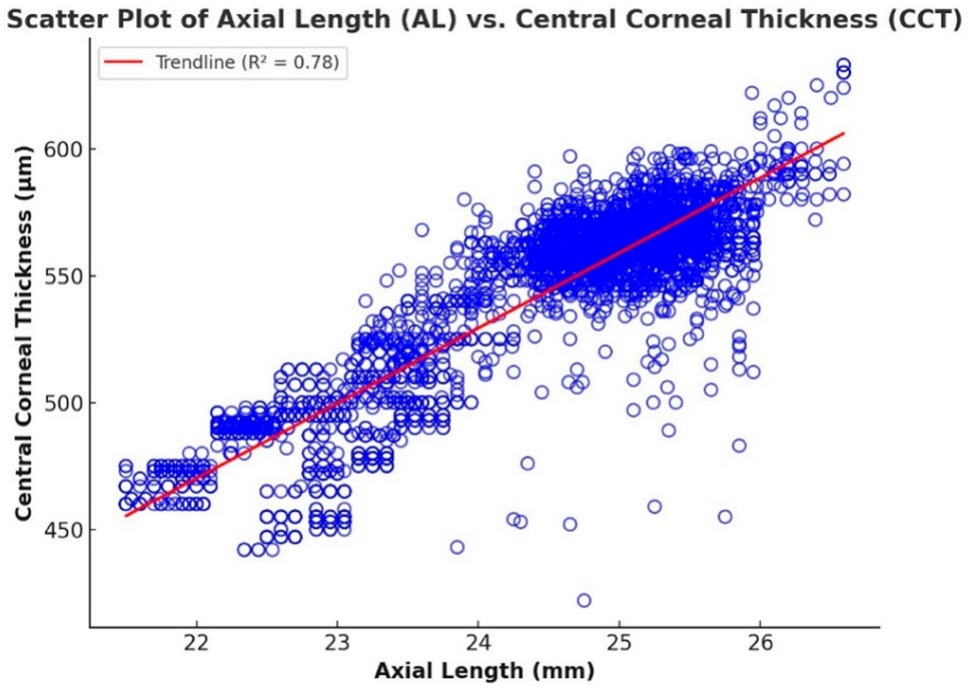
Scatter plot showing a correlation between axial length and central corneal thickness

**Fig. 4:**
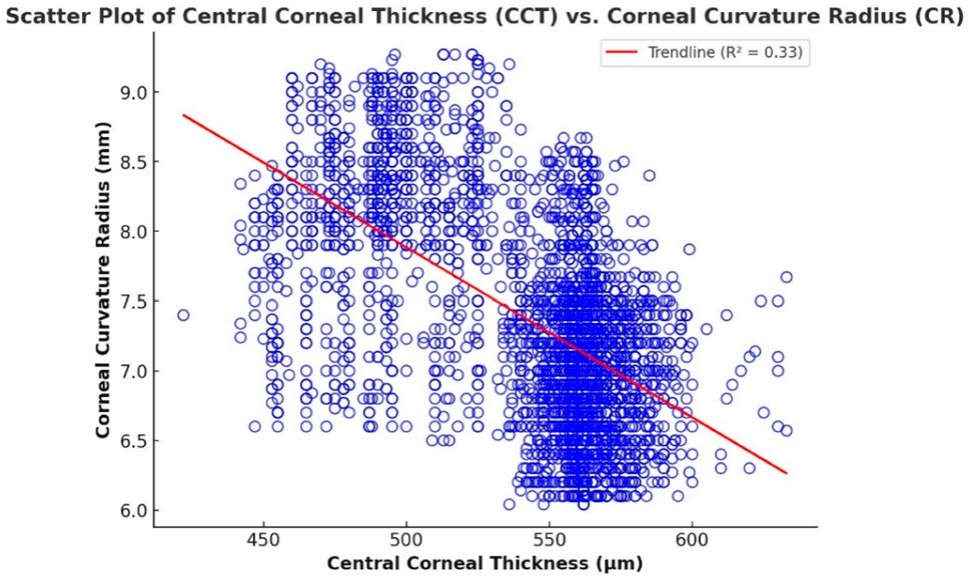
Scatter plot showing a correlation between corneal radius and central corneal thickness

**Fig. 5:**
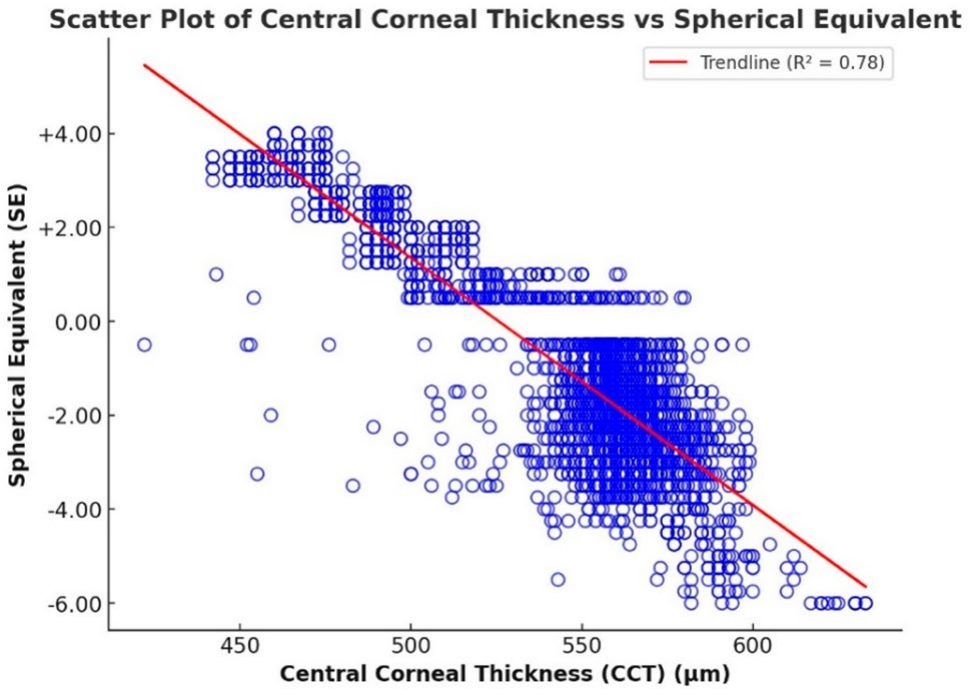
Scatter plot showing a correlation between SE and central corneal thickness

**Fig. 6:**
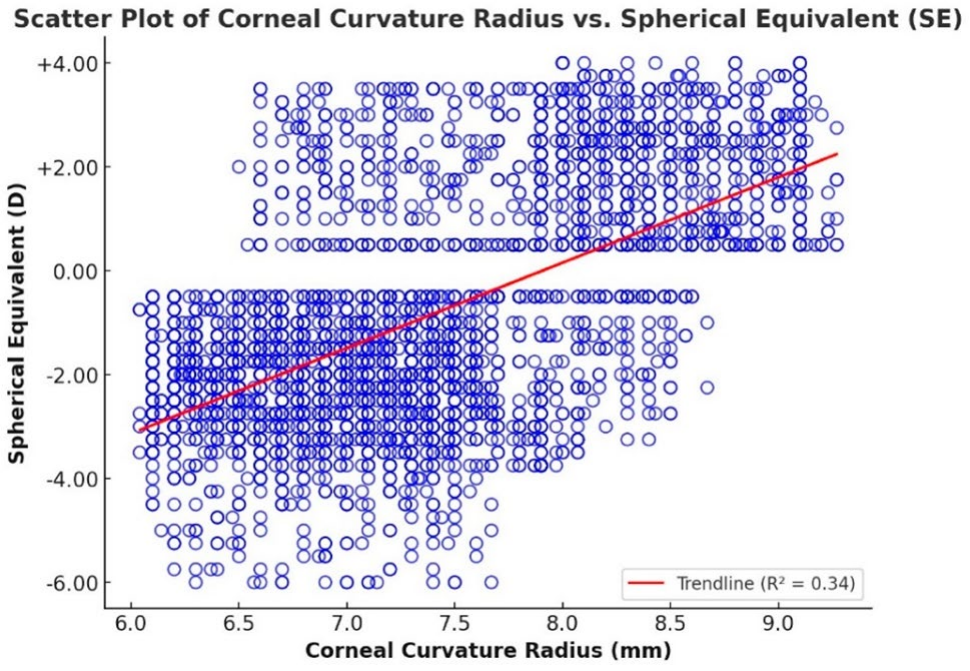
Scatter plot showing a SE and corneal radius (CR) correlation

To investigate the relationships among key ocular biometric parameters and refractive errors, both bivariate correlation and multiple linear regression analyses were employed. Initially, scatter plots were generated to visually assess the interactions between axial length (AL), corneal radius (CR), central corneal thickness (CCT), and the spherical equivalent (SE), which serves as a quantitative representation of refractive error. These visual and statistical examinations were conducted across multiple pairwise combinations, as presented in Figs. 1, 2, 3, 4, 5 and 6.

Figure 2 depicts a statistically significant inverse correlation between axial length (AL) and corneal radius (CR), with a Pearson correlation coefficient of *r* = -0.63 (*p* < 0.001). This finding suggests that eyes with increased axial length, typically associated with myopic refractive status, tend to have steeper corneas, as reflected by smaller CR values. The increased curvature of the cornea contributes to enhanced refractive power, thus reinforcing the progression of myopia.

To further quantify the predictive contribution of AL and CR to refractive outcomes, a multiple linear regression analysis was performed. The results revealed that both AL and CR are statistically significant predictors of the spherical equivalent (SE), with AL exerting a markedly stronger effect (β = -2.0035) than CR (β = -0.1482). The final regression model is as follows:

$$\text{SE}=49.1778-2.0035\times\left(\text{AL}\right)-0.1482\times\left(\text{CR}\right)$$

This model accounts for 94.5% of the variance in SE (R² = 0.945), indicating excellent explanatory power. These findings underscore the dominant role of axial elongation in the development of myopia, while also affirming the independent but less pronounced contribution of corneal curvature. Specifically, steeper corneas (smaller CR values) are associated with myopia, whereas flatter corneas (larger CR values) are more often observed in hyperopic eyes. Together, AL and CR offer a comprehensive predictive framework for understanding refractive status in young adults.

Figure 3 illustrates a strong positive correlation between axial length (AL) and central corneal thickness (CCT), with a Pearson correlation coefficient of *r* = 0.885 (*p* < 0.001). This statistically significant relationship suggests that eyes with greater axial elongation tend to exhibit thicker central corneas. While the precise physiological mechanism remains to be fully elucidated, this trend may reflect a compensatory structural adaptation aimed at preserving corneal integrity and biomechanical stability in the context of increased ocular length.

To further investigate the predictive contributions of these parameters to refractive status, a multiple linear regression analysis was conducted with spherical equivalent (SE) as the dependent variable. The model revealed that both AL and CCT are significant predictors of SE (*p* < 0.001), with AL demonstrating a substantially stronger effect (β = -2.0035) compared to CCT (β = -0.1482). The regression equation is as follows:

$$\text{SE}=49.1778-2.0035\times\left(\text{AL}\right)-0.1482\times\left(\text{CCT}\right)$$

This model accounts for 78.2% of the variance in SE (R² = 0.782), indicating a robust explanatory capacity. The negative coefficient for AL confirms that axial elongation is closely associated with increasing myopia (more negative SE values), while the additional contribution of CCT, although statistically significant, exerts a comparatively modest effect. These findings reinforce the dominant role of AL in myopia development, while highlighting the potential biomechanical relevance of CCT in supporting the structural demands imposed by axial elongation. Thus, the interplay between ocular length and corneal architecture should be considered a key element in the pathophysiology of refractive errors.

Figure 4 demonstrates a moderate negative correlation between central corneal thickness (CCT) and corneal curvature radius (CR), with *r* = -0.577 (*p* < 0.001). This indicates that thinner corneas are generally associated with flatter profiles (larger CR values), while thicker corneas tend to be steeper. The observed relationship may reflect a biomechanical interaction, whereby corneal structure and shape adjust in tandem to maintain optical performance.

Complementing this, multiple linear regression analysis confirmed that both CCT and CR are significant predictors of the spherical equivalent (SE). The regression equation is:

$$\text{SE}=23.3048-0.0488\times\left(\text{CCT}\right)+0.3110\times\left(\text{CR}\right)$$

The model explains 33.3% of the variance in SE (R² = 0.333), with CCT negatively associated and CR positively associated with SE. These findings suggest that increased corneal thickness contributes to more myopic refractive outcomes, while flatter corneas are linked to hyperopia. Together, CCT and CR offer moderate but meaningful insight into refractive error variation.

Figure 5 illustrates a strong inverse relationship between central corneal thickness (CCT) and spherical equivalent (SE), with a correlation coefficient of *r* = -0.883 (*p* < 0.001). This suggests that individuals with thicker central corneas are more likely to present with greater degrees of myopia. This association may reflect an underlying biomechanical adaptation, whereby increased corneal thickness potentially contributes to structural stability in eyes undergoing axial elongation.

To further quantify this relationship, a simple linear regression analysis was performed, producing the following predictive equation:

$$\text{SE}=27.6523-0.0526\times\left(\text{CCT}\right)$$

The model explains approximately 77.9% of the variance in SE (R² = 0.779), indicating that CCT alone is a substantial predictor of refractive error. The negative regression coefficient confirms that greater CCT is associated with more myopic outcomes. These findings underscore the relevance of corneal thickness not only as a biometric parameter but also as a potential contributor to refractive development, particularly in highly myopic eyes where mechanical stability is crucial.

Figure 6 displays a moderate positive correlation between corneal curvature radius (CR) and spherical equivalent (SE), with *r* = 0.583 (*p* < 0.001) and R² = 0.34. This indicates that as the cornea becomes flatter (i.e., larger CR values), SE tends to increase, reflecting a shift toward hyperopic refractive status.

To further evaluate this relationship, a simple linear regression analysis was performed, yielding the following model:

$$\text{SE}=-13.0045+1.6450\times\left(\text{CR}\right)$$

The model explains 34% of the variance in SE, supporting the role of corneal curvature in influencing refractive error. The positive regression coefficient confirms that flatter corneas are associated with more hyperopic outcomes, while steeper corneas (smaller CR) are linked to myopia. These findings emphasize the optical significance of CR as an independent contributor to refractive power and reinforce its relevance in both clinical evaluation and refractive error modeling.

### Gender differences in biometric parameters

**Fig. 7:**
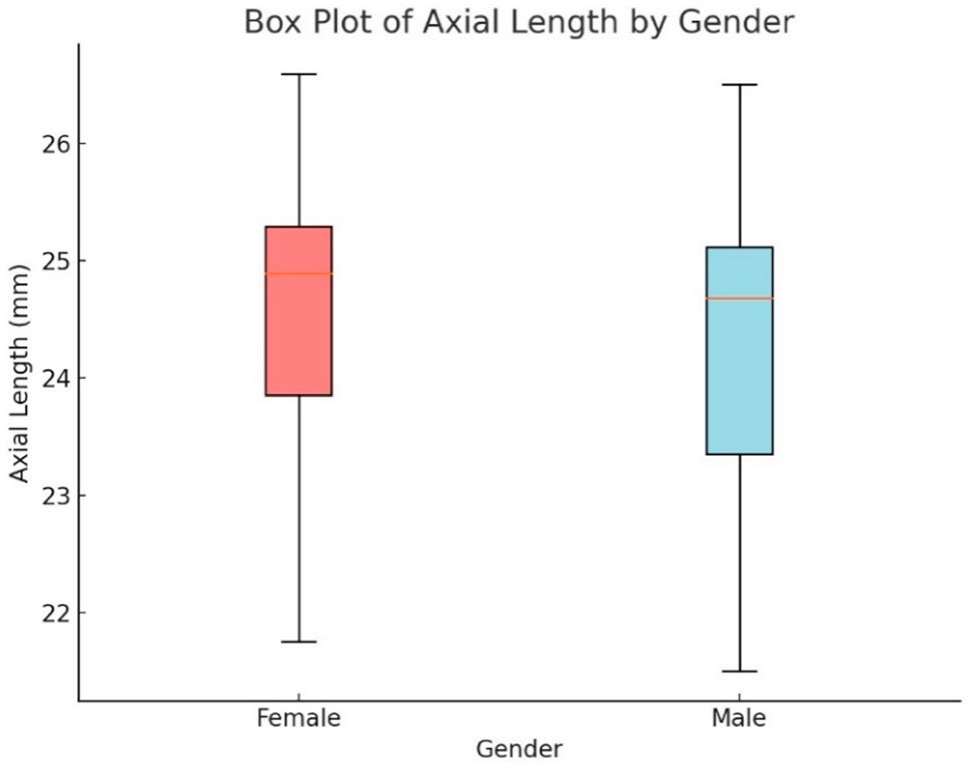
Boxplot of axial length by gender

Figure 7 illustrates the distribution of axial lengths (AL) by sex, showing that females generally have longer axial lengths (25.35 ± 0.50 mm) compared to males (24.95 ± 0.61 mm). This difference was statistically significant (*p* < 0.001) based on the results of an independent sample t-test, which was conducted to compare the means of AL between the two gender groups.

This analysis indicates a clear gender difference in axial length, with females consistently exhibiting longer AL than males. The significant p-value further supports the hypothesis that gender plays a role in the variation of axial length, a key biometric parameter influencing refractive errors.

### Prevalence of refraction errors and ocular biometric parameters by gender

**Fig. 8:**
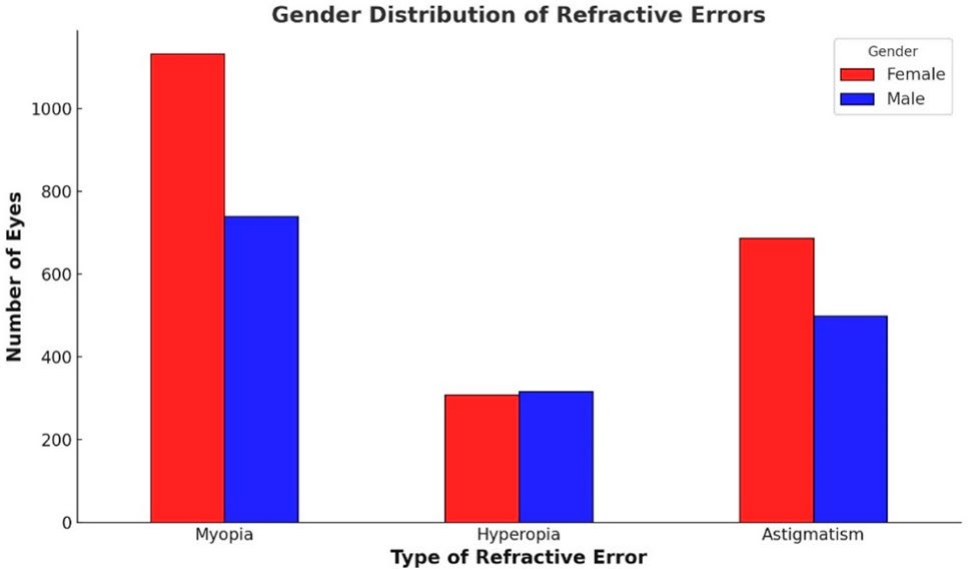
Distribution of patients’ eyes by type of refractive error

Figure 8 illustrates the prevalence of refraction errors categorized by sex. Myopia and astigmatism were more prevalent in females, while hyperopia showed a similar distribution across sexes.

### Ocular biometric differences by refractive error group

**Fig. 9:**
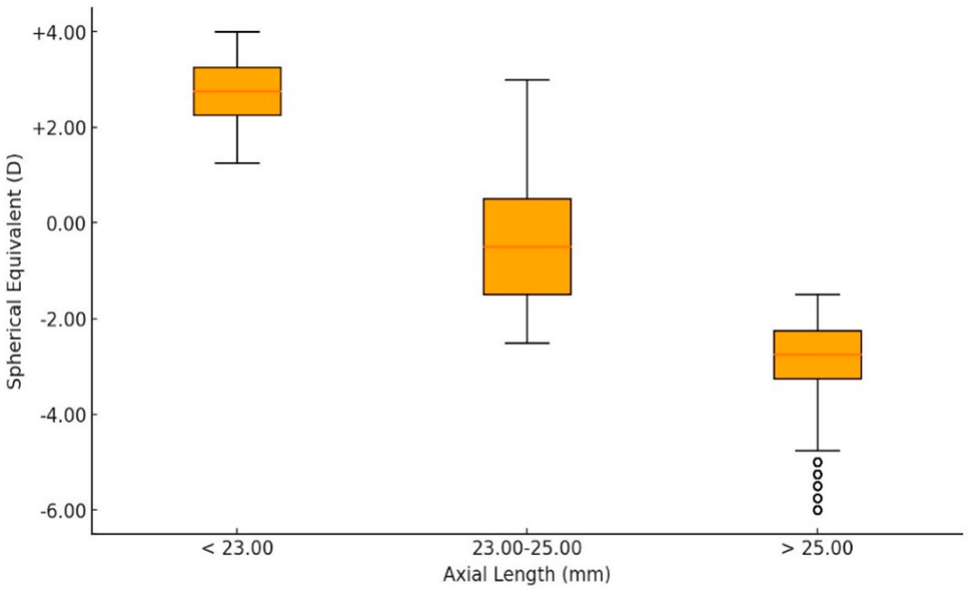
Boxplot of axial length (AL) by spherical equivalent (SE)

Figure 9 shows the eyes with shorter ALs (< 23 mm) were predominantly hyperopic, while longer ALs (> 25 mm) were associated with myopia.

**Fig. 10:**
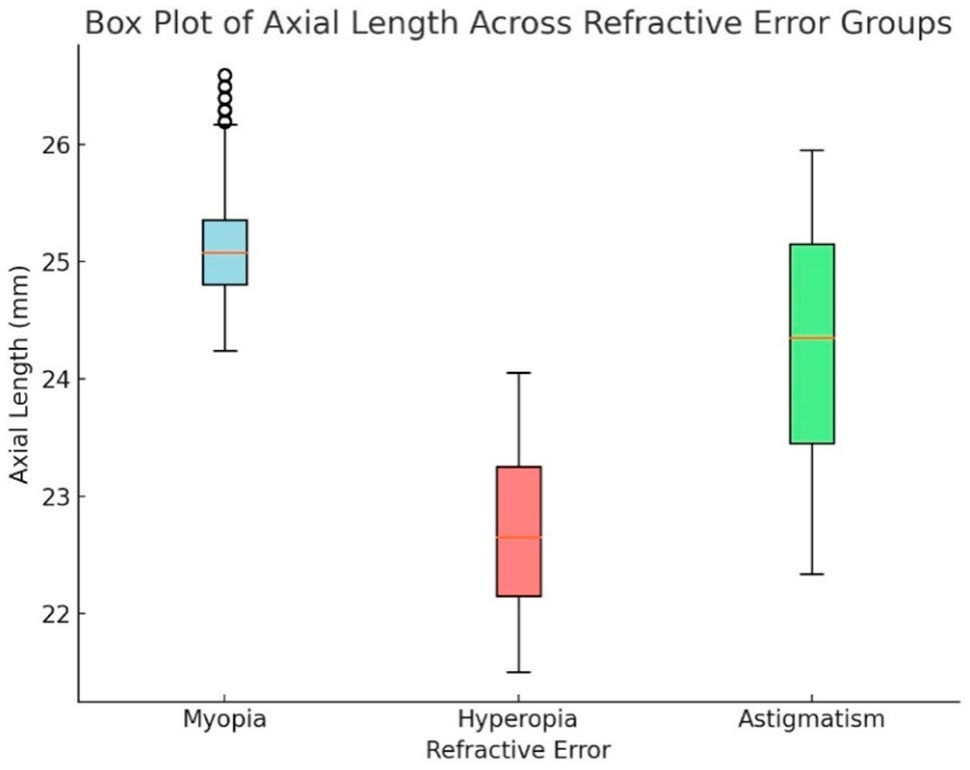
Boxplot of axial length across refractive error groups

Figure 10 shows the myopic eyes had the longest ALs (24–26 mm), while hyperopic eyes had the shortest (< 23 mm).

### Central corneal thickness and corneal radius across refractive error groups

**Fig. 11:**
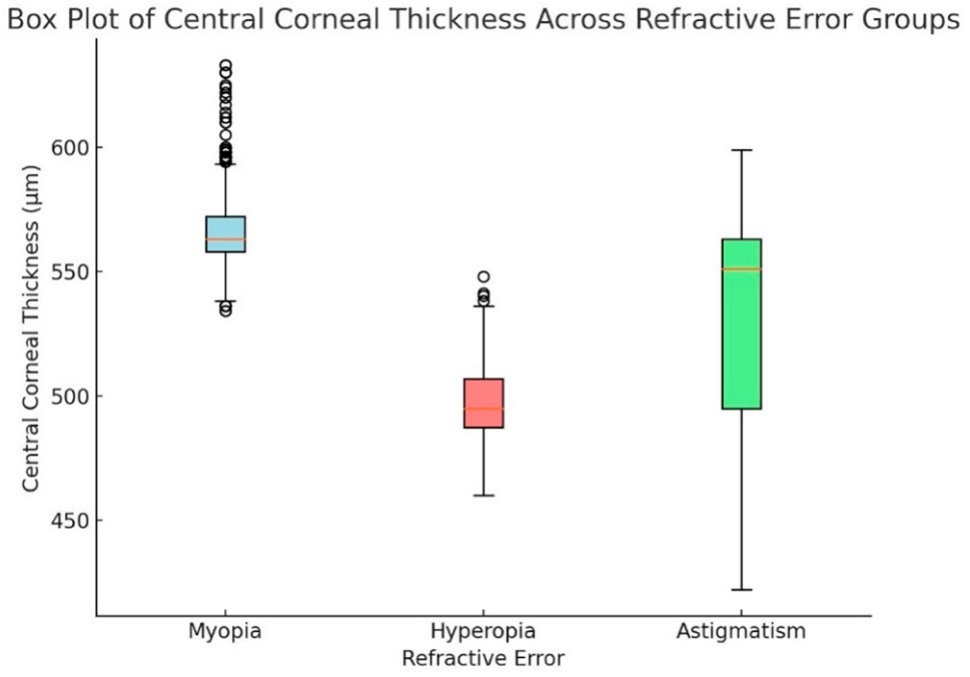
Box plot central corneal thickness across Refractive Error Groups

Figure 11 shows the myopic eyes had the thickest CCT (550–600 μm), while hyperopic eyes had the thinnest (< 500 μm).

**Fig. 12:**
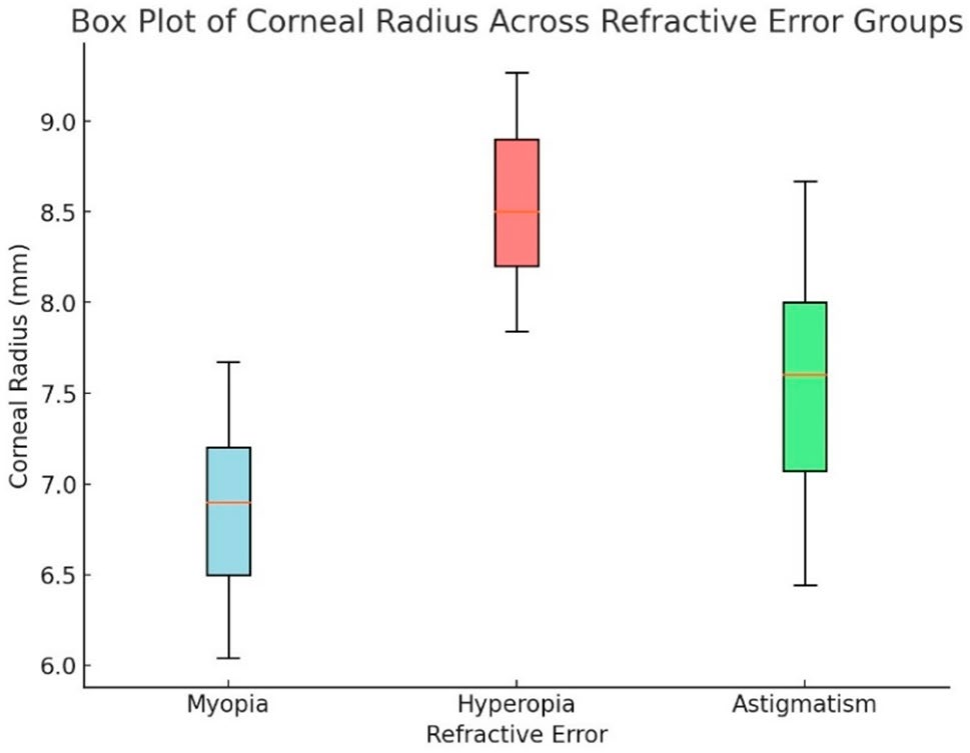
Box plot of corneal radius across refractive error groups

Figure 12 shows the myopic eyes had the steepest corneas (6.5–7.5 mm CR), and hyperopic eyes had the flattest (8.0–9.0 mm CR).

**Fig. 13:**
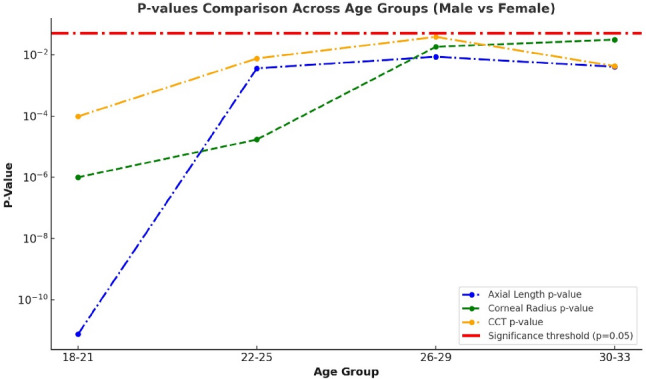
P-value comparison between males and females by age groups

Figure 13 shows the p-values for axial length, corneal radius, and central corneal thickness across different age groups. The p-values for axial length are particularly low in the 18–21 age group, indicating significant variation in axial length with age. For the corneal radius, the p-values remain below the significance threshold but increase with age. The statistical significance of central corneal thickness decreases with age but remains relevant.

### Analysis of biometric ocular parameters across refractive error groups and gender differences

Ocular biometric characteristics axial length, corneal radius, and central corneal thickness are compared across myopic, hyperopic, and astigmatic refractive error groups. A gender-based breakdown is also provided to analyze differences between male and female participants within each refractive error group.

Table 2 presents the findings of the one-way analysis of variance (ANOVA) for biometric ocular parameters across refractive error categories (*p* < 0.001).

**Table 2:** One-way ANOVA for biometric ocular parameters among refractive error groups

Parameter	F-statistic	*p*-value
Axial Length	F(2, 3697) = 2967	*p* < 0.001
Corneal Curvature	F(2, 3697) = 2936	*p* < 0.001
Central Corneal Thickness	F(2, 3697) = 1944	*p* < 0.001

Table 3 compares axial length (AL) between males and females within each refractive error group. Females consistently exhibit significantly longer ALs than males across all groups (*p* < 0.0001).

**Table 3:** Mean axial length by refractive error and gender

Refractive Error	Male			Female			p-value
	N	Mean ± SD	95% CI	N	Mean ± SD	95% CI	
Myopia	739	25.04 ± 0.42	25.01–25.07	1132	25.15 ± 0.41	25.13–25.18	< 0.0001
Hyperopia	317	22.60 ± 0.64	22.53–22.68	309	22.82 ± 0.64	22.74–22.89	< 0.0001
Astigmatism	499	24.17 ± 0.96	24.09–24.26	686	24.42 ± 0.95	24.35–24.49	< 0.0001

95% CI − 95% confidence interval

Table 4 presents axial length comparisons within each refractive error group and overall, using the LSD method. Significant differences were observed: myopic participants had a greater axial length than astigmatic participants (mean difference: 0.794 mm, *p* < 0.0001) and hyperopic participants (mean difference: 2.399 mm, *p* < 0.0001). Astigmatic eyes also exhibited a longer axial length than hyperopic eyes (mean difference: 1.606 mm, *p* < 0.0001).

**Table 4:** Pairwise comparisons of axial length by refractive error groups

Group 1	Group 2	Mean Difference	95% CI	*p*-value
Myopia	Astigmatism	0.794	0.736–0.852	< 0.0001
Myopia	Hyperopia	2.399	2.345–2.454	< 0.0001
Astigmatism	Hyperopia	1.606	1.531–1.681	< 0.0001

Table 5 summarizes axial length (AL) by sex and spherical equivalent (SE) range, showing significant sex differences across all SE groups. Females consistently exhibit longer AL than males, including in the SE range − 0.50 to -2.00 (females: 24.82 ± 0.22 mm; males: 24.64 ± 0.25 mm, *p* < 0.0001), -2.25 to -4.00 (females: 25.44 ± 0.23 mm; males: 25.28 ± 0.20 mm, *p* < 0.0001), and − 4.25 to -6.00 (females: 26.13 ± 0.29 mm; males: 25.95 ± 0.25 mm, *p* = 0.0003).

**Table 5:** Mean axial length (AL) by spherical equivalent (SE) range and sex

Group	SE	Male			Female			
		*N*	Mean AL ± SD	95% CI	*N*	Mean AL ± SD	95% CI	*p*-value
1	-0.50 to -2.00	519	24.64 ± 0.25	24.62–24.66	769	24.82 ± 0.22	24.81–24.84	< 0.0001
2	-2.25 to -4.00	456	25.28 ± 0.20	25.26–25.30	666	25.44 ± 0.23	25.43–25.46	< 0.0001
3	-4.25 to -6.00	53	25.95 ± 0.25	25.89–26.02	72	26.13 ± 0.29	26.06–26.20	= 0.0003
4	+ 0.50 to + 2.00	280	23.31 ± 0.38	23.26–23.35	350	23.57 ± 0.43	23.52–23.61	< 0.0001
5	+ 2.25 to + 4.00	247	22.32 ± 0.46	22.26–22.38	270	22.64 ± 0.50	22.58–22.70	< 0.0001

## Discussion

This study clarifies the relationship between ocular biometric parameters and refractive errors among university-aged participants in Iraq. A significant association was observed between axial length (AL) and refractive status, with myopic individuals displaying longer ALs than hyperopic or emmetropic counterparts. This finding is consistent with previous studies, including Fan et al. [36] in Chinese university students and Hashemi et al. [11] in the Tehran Geriatric Eye Study, affirming axial elongation as a key structural contributor to myopia.

The gender differences observed in our study have implications for clinical practice and public health policies. Females exhibited longer axial lengths across all refractive error categories and spherical equivalent (SE) ranges compared to males, particularly in high myopia cases (e.g., SE − 4.25 to − 6.00, AL = 26.13 ± 0.29 mm in females vs. 25.95 ± 0.25 mm in males; *p* = 0.0003). These observations differ from Fan et al. [36], who reported longer ALs in Chinese males. This contrast suggests regional or genetic factors, hormonal influences, and environmental exposures, such as increased near-work and less outdoor activity among females, may contribute to axial elongation. Similar gender-related findings were reported by Zeried et al. [19] in a Saudi population, reinforcing the need for sex-specific approaches in refractive screening and intervention.

In addition to AL, the study demonstrated significant associations between central corneal thickness (CCT) and refractive error. Myopic eyes tended to have thicker corneas, while hyperopic eyes had thinner ones. The negative correlation between CCT and SE (*r* = − 0.883, *p* < 0.001) suggests that increased CCT may serve as a biomechanical adaptation to axial elongation, providing structural support to maintain ocular integrity. This observation aligns with results from the Andhra Pradesh Eye Disease Study (APEDS) in South India, where myopic individuals were found to have significantly thicker corneas, and the prevalence of myopia was higher among younger adults and females [37].

Furthermore, corneal curvature radius (CR) was moderately associated with SE (*r* = 0.583), with flatter corneas (larger CR) corresponding to more hyperopic outcomes. Regression analyses confirmed that both CR and CCT independently predict SE, though AL remains the dominant factor. Notably, CCT and CR also exhibited a moderate inverse correlation (*r* = − 0.577), suggesting a structural interaction in shaping refractive power. These findings emphasize the importance of considering CCT and CR jointly with AL when assessing refractive status.

However, the relationship between CCT and refractive error is not universally consistent. Almazrou et al. [37] found no significant difference in CCT between myopic and non-myopic individuals, indicating that this parameter may vary across populations. Nevertheless, the present study’s results are supported by other reports, including Khoramnia et al. [7], who highlighted the clinical importance of CCT in refractive surgery outcomes, and Alrashidi [38], who demonstrated its role in guiding photorefractive keratectomy in Saudi patients.

Age-related thinning of CCT has also been widely documented, attributed to changes in collagen density and hydration within the corneal stroma. Although our sample consisted of young adults, future research could extend these findings across a broader age spectrum to evaluate refractive stability and its interaction with corneal biomechanics over time.

The results of this study require more investigation, particularly concerning the lasting influence of genetic and environmental factors on gender differences in AL. The hypothesized importance of the ocular surface bacteria in the emergence of refractive error necessitates additional research [39]. Recent studies suggest that an imbalance in the ocular surface microbiome may trigger inflammatory responses and biomechanical degradation of the cornea, particularly influencing parameters such as central corneal thickness (CCT) and corneal curvature (CR). These abnormalities may exacerbate myopia progression or result in conditions like as keratoconus, which is marked by anomalies in corneal curvature. Given the increasing prevalence of myopia and its association with environmental and lifestyle factors, more investigation into the ocular microbiome may provide valuable insights into the pathogenesis of refractive defects and uncover novel therapeutic or preventive strategies. Moreover, further research is required to examine the relationship between CCT and refractive outcomes in post-surgical patients, since this may significantly impact long-term treatment strategies [40].

This study has limitations. The cross-sectional design restricts causal inference, and the university-based sample may not reflect broader demographic variability. However, the large sample size, robust statistical modeling, and integration of biometric variables strengthen the validity of our findings.

In conclusion, our research underscores the importance of biometric ocular factors such as axial length, corneal curvature, and central corneal thickness in evaluating refractive status. These findings corroborate and enhance prior research carried out in nearby regions and globally. Our findings suggest that female students may be more susceptible than male students to severe refractive errors, particularly myopia, underscoring the necessity for gender-sensitive strategies for addressing this problem. As urbanization and lifestyle changes continue to impact refractive error prevalence in the Middle East and worldwide, our findings contribute valuable knowledge that can inform public health and clinical strategies for addressing this significant health concern. This study’s insights have direct applications in patient management and public health, as uncorrected refractive errors remain one of the leading causes of visual impairment globally.

## Electronic supplementary material

Below is the link to the electronic supplementary material.

Supplementary Material 1

## Data Availability

The date that supports the findings of this study are available in the supplementary material of this article.
